# PCDA–EDA Colorimetric Nanofiber Sensor for Rapid Visual GHB Screening: Linker Reassignment and Scalable Fabrication

**DOI:** 10.3390/bios16080440

**Published:** 2026-08-14

**Authors:** Seunghye Yang, Jeongwook Lee, Om Darlami, Dongyun Shin

**Affiliations:** College of Pharmacy, Gachon University, 191 Hambakmoe-ro, Yeonsu-gu, Incheon 21936, Republic of Korea; 202055502@gachon.ac.kr (S.Y.); ljw7764@chemone.kr (J.L.)

**Keywords:** gamma-hydroxybutyric acid, polydiacetylene, colorimetric sensor, electrospun nanofiber mat, paper-based sensor, supramolecular recognition

## Abstract

γ-Hydroxybutyric acid (GHB), a colorless and odorless central nervous system depressant associated with drug-facilitated sexual assault, demands rapid on-site detection. Polydiacetylene (PDA) colorimetric sensors derived from 10,12-pentacosadiynoic acid (PCDA) conjugates are a promising platform, but the molecular origin of GHB recognition in PCDA–gabazine systems has remained unresolved. Here, we compare a series of structurally related PCDA conjugates to examine how the chemical state of the EDA-derived unit affects the GHB-induced colorimetric response. A side-by-side substituent screen of three PCDA derivatives showed that PCDA–EDA produced the largest colorimetric response (ΔR = 53) within 30 s, the hydrazide analogue gave a moderate response (ΔR = 34), and a simple amide was negligible (ΔR = 12). By contrast, a PCDA–gabazine mat prepared by the same protocol showed only a subtle, barely discernible color shift after several hours and remained predominantly blue even after approximately 24 h, without a visually appreciable blue-to-red transition. This difference suggests that the accessible free primary amine of PCDA–EDA is an important factor contributing to its faster and stronger response. Building on this mechanistic finding, we replaced the previously used PVDF–HFP/PEO matrix with a cellulose/PVDF–HFP formulation processed from DMF and adopted multi-nozzle electrospinning, reducing the fabrication time from approximately 120 to 30 min per sheet, corresponding to a 75% reduction in processing time. The sensor mat showed a clearly distinguishable, dose-dependent visual response across 0.5–3% *w*/*v* GHB within 30 s, covering the forensically relevant concentration window. These findings reposition linker architecture as a central design parameter for PDA-based forensic colorimetric sensors.

## 1. Introduction

Clandestine administration of γ-hydroxybutyric acid (GHB) poses persistent public safety and forensic challenges worldwide. As a potent central nervous system depressant that is colorless and odorless in beverages, GHB is widely implicated in drug-facilitated sexual assault and related crimes [1]. Its rapid metabolism and short detection window mean that cases are routinely underreported or confirmed only by retrospective toxicology, underscoring the need for rapid, reliable, field-deployable detection [2].

Gas chromatography–mass spectrometry and liquid chromatography–tandem mass spectrometry remain the analytical gold standards for forensic GHB quantification because of their sensitivity and specificity [2]. However, their dependence on sample preparation, expensive instrumentation, and trained personnel restricts them from point-of-use screening, motivating active research into low-cost, portable sensing platforms [3,4,5,6,7].

**Figure 1:**
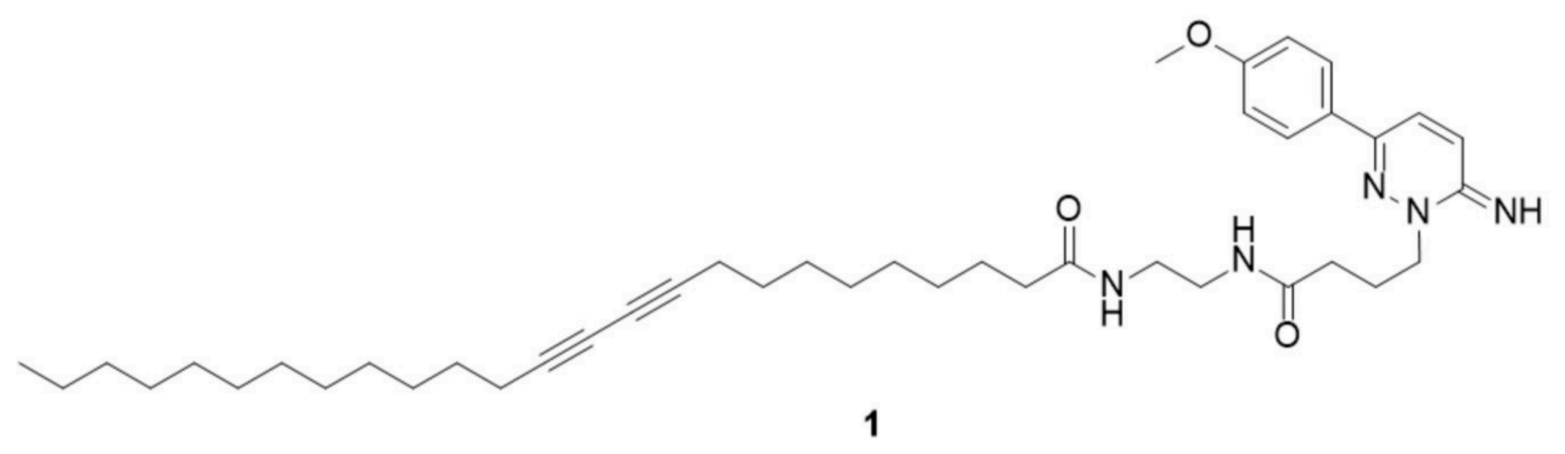
Chemical structure of PCDA–gabazine (**1**).

Colorimetric sensors based on polydiacetylene (PDA), a conjugated polymer formed by 254-nm photopolymerization of diacetylene monomers such as 10,12-pentacosadiynoic acid (PCDA), are particularly attractive in this context. The conjugated ene-yne backbone of PDA undergoes a distinctive blue-to-red chromatic transition in response to environmental and molecular perturbations, providing a direct visual readout without external instrumentation [8,9,10,11,12]. Several PCDA-conjugate sensors for GHB have been reported, the most prominent of which incorporate a gabazine pharmacophore tethered to PCDA via an ethylenediamine (EDA) bridge (Figure 1). Recognition has generally been attributed to the gabazine moiety [11,13]; however, this assignment has rested on functional inference rather than direct experimental comparison of the gabazine pharmacophore against the linker itself.

Recent observations across related supramolecular sensor systems suggest that linker architecture—specifically, the electronic and steric environment around the amine bridge—can dominate analyte affinity and chromatic response [13,14]. This raises a basic mechanistic question for PCDA-based GHB recognition: is the gabazine pharmacophore actually doing the work, or is the EDA linker the true recognition site?

Herein, we resolve this question and translate the answer into a practical visual screening device. We (i) synthesize three PCDA derivatives—PCDA–amide (**3**), PCDA–hydrazide (**4**), and PCDA–EDA (**5**)—and compare their colorimetric response to GHB head-to-head, including a direct comparison with a PCDA–gabazine mat prepared by the same protocol; (ii) modify the sensor matrix formulation and electrospinning process to reduce fabrication time; and (iii) establish an improved synthetic route to PCDA–gabazine to secure its supply for future comparative work. The experimental results suggest that the chemical state and accessibility of the EDA-derived amine functionality strongly influence the GHB-induced colorimetric response. Accordingly, linker architecture should be considered alongside pharmacophore structure in the design of PDA-based colorimetric sensors. This finding repositions linker architecture as a central—rather than ancillary—design parameter for PDA-based colorimetric sensors.

## 2. Experimental Section

### 2.1. Materials and Instrumentations

10,12-Pentacosadiynoic acid (PCDA) was purchased from Bide Pharmatech Ltd. (Shanghai, China). *N*-Hydroxysuccinimide (NHS), ethylenediamine (EDA), triethylamine (TEA), dichloromethane (DCM), *N*,*N*-dimethylformamide (DMF), N-methyl-2-pyrrolidone (NMP), and tert-butyl (2-aminoethyl)carbamate were purchased from Sigma-Aldrich (St. Louis, MO, USA). 1-Ethyl-3-(3-dimethylaminopropyl)carbodiimide hydrochloride (EDC) was purchased from Thermo Fisher Scientific (Waltham, MA, USA). γ-Hydroxybutyric acid (GHB) was supplied by Sam Eung Industrial Co., Ltd. (Seoul, Korea) under official permission from the Korean Ministry of Food and Drug Safety (permission no. Gyeongin-995). All other reagents were of analytical grade and used as received.

^1^H NMR spectra were recorded on a Bruker 600 MHz spectrometer (Bruker, Billerica, MA, USA) in CDCl_3_ or DMSO-*d*_6_. Chemical shifts are reported in ppm (δ) relative to the residual solvent signal, with coupling constants (*J*) in Hz. Multiplicities are abbreviated as follows: s = singlet, d = doublet, t = triplet, q = quartet, p = pentet, sept = septet, m = multiplet, br = broad. The 1H NMR spectra and corresponding peak assignments of the synthesized compounds are provided in the Supporting Information. Microwave-assisted reactions were performed using an Anton Paar Monowave 300 microwave synthesis reactor (Anton Paar GmbH, Graz, Austria) with a maximum microwave output power of 850 W.

### 2.2. Synthesis of PCDA Derivatives

**Scheme 1:**
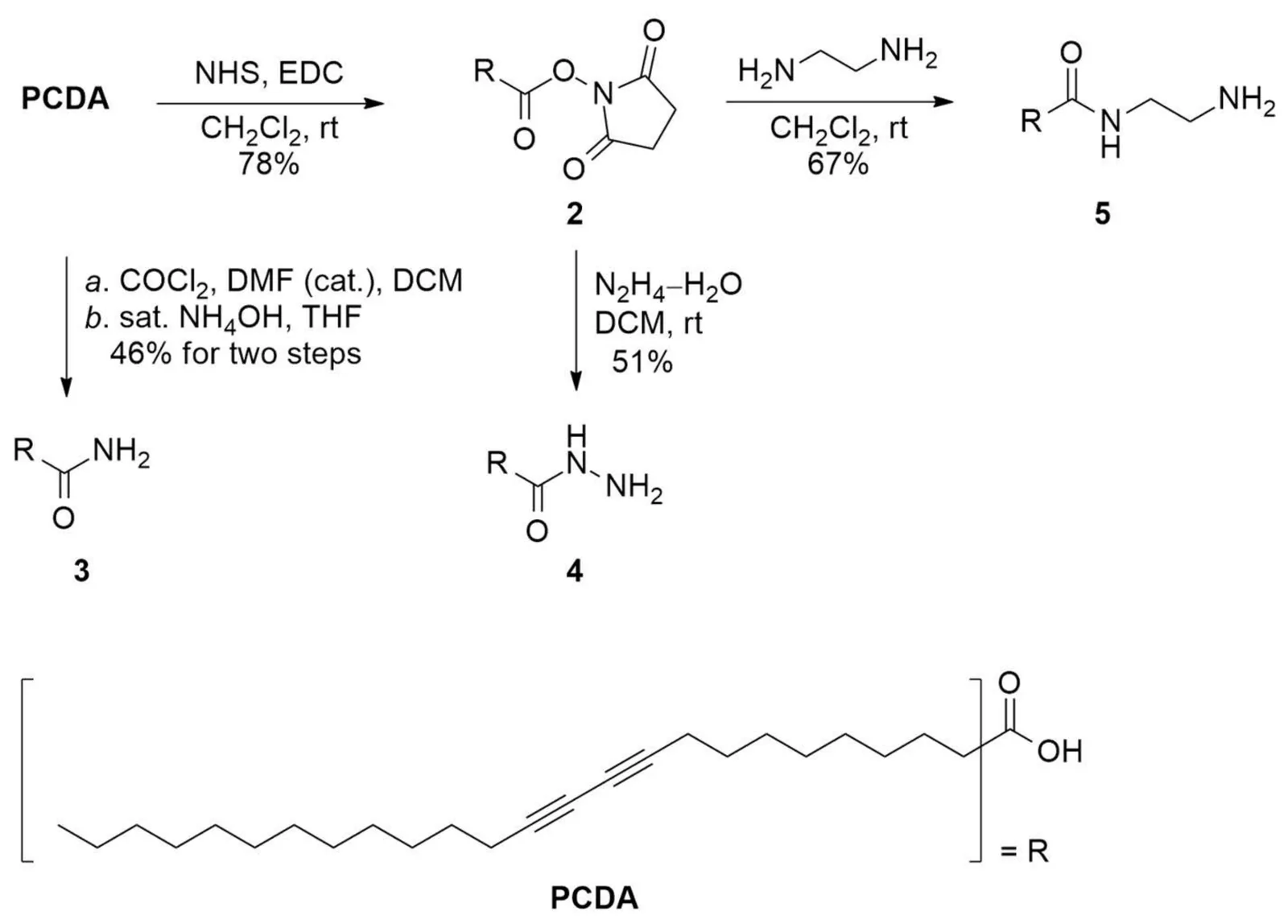
Synthesis of PCDA derivatives (**2**–**5**).

PCDA derivatives **3**–**5** were prepared from PCDA as outlined in Scheme 1. Activation of PCDA with EDC/NHS afforded the *N*-hydroxysuccinimidyl ester **2**, which was reacted separately with ethylenediamine and hydrazine hydrate to yield PCDA–EDA (**5**) and PCDA–hydrazide (**4**), respectively. PCDA–amide (**3**) was prepared independently by converting PCDA to the corresponding acyl chloride with oxalyl chloride and quenching with aqueous ammonia.

#### 2.2.1. PCDA–NHS (**2**)

EDC (620 mg, 4.0 mmol) and NHS (460 mg, 4.0 mmol) were added to a solution of PCDA (1.00 g, 2.67 mmol) in anhydrous DCM (15 mL) at 0 °C. The mixture was stirred overnight at room temperature. Volatiles were removed in vacuo; the residue was redissolved in EtOAc, and the organic layer was washed with brine and water, dried over anhydrous MgSO_4_, filtered, and concentrated. Purification by silica gel chromatography (DCM/MeOH, 25:1) afforded **2** as a white solid (960 mg, 78%). ^1^H NMR (600 MHz, CDCl_3_) δ 2.84 (d, *J* = 7.9 Hz, 4H), 2.60 (td, *J* = 7.5, 1.3 Hz, 2H), 2.27–2.21 (m, 4H), 1.74 (td, *J* = 7.6, 1.4 Hz, 2H), 1.51 (p, *J* = 7.2 Hz, 4H), 1.43–1.35 (m, 6H), 1.34–1.22 (m, 21H), 0.88 (td, *J* = 7.1, 1.4 Hz, 3H).

#### 2.2.2. PCDA–Amide (**3**)

Oxalyl chloride (0.15 mL, 1.7 mmol) and two drops of DMF were added to a solution of PCDA (500 mg, 1.3 mmol) in anhydrous DCM at 0 °C. The mixture was stirred overnight at room temperature, and the volatiles were removed under reduced pressure to give 10,12-pentacosadiynoyl chloride as a yellow oil, which was used directly. The acyl chloride was dissolved in THF and added dropwise to aqueous ammonium hydroxide (28–30%, 1.3 equiv) at 0 °C. After stirring overnight at room temperature, water was added and the mixture was extracted with DCM. The combined organic layers were washed with brine, dried over anhydrous MgSO_4_, filtered, and concentrated in vacuo to give **3** as a white solid (250 mg, 46% over two steps). ^1^H NMR (600 MHz, CDCl_3_) δ 5.40 (s, 2H), 2.23 (dt, *J* = 12.6, 7.3 Hz, 6H), 1.66–1.60 (m, 2H), 1.54–1.48 (m, 4H), 1.40–1.22 (m, 26H), 0.89 (t, *J* = 7.0 Hz, 3H).

#### 2.2.3. PCDA–Hydrazide (**4**)

Hydrazine hydrate (0.10 mL, 3.18 mmol) was added to a solution of PCDA–NHS (**2**) (500 mg, 1.06 mmol) in DCM (10 mL). The mixture was stirred at room temperature for 12 h. The combined organic layers were washed twice with water, dried over anhydrous MgSO_4_, filtered, and concentrated in vacuo to afford **4** as a white solid (210 mg, 51%). ^1^H NMR (600 MHz, CDCl_3_) δ 6.67 (s, 1H), 3.89 (d, *J* = 4.2 Hz, 2H), 2.24 (t, *J* = 7.0 Hz, 4H), 2.17–2.12 (m, 2H), 1.66–1.60 (m, 2H), 1.54–1.48 (m, 4H), 1.40–1.34 (m, 4H), 1.28 (br, 23H), 0.88 (t, *J* = 7.0 Hz, 3H).

#### 2.2.4. PCDA–EDA (**5**)

PCDA–NHS (**2**) (630 mg, 1.33 mmol) in DCM was added dropwise to a stirred mixture of ethylenediamine (0.90 mL, 13.35 mmol) in DCM (20 mL). The mixture was stirred at room temperature for 12 h. The organic phase was separated and washed twice with water, then with brine, dried over anhydrous MgSO_4_, filtered, and concentrated in vacuo to give **5** as a white solid (370 mg, 67%). ^1^H NMR (600 MHz, CDCl_3_) δ 5.89 (s, 1H), 3.30 (q, *J* = 5.8 Hz, 2H), 2.85–2.81 (m, 2H), 2.26–2.22 (m, 4H), 2.20–2.16 (m, 2H), 1.66–1.60 (m, 2H), 1.51 (dtd, *J* = 12.6, 7.1, 4.0 Hz, 4H), 1.40–1.34 (m, 4H), 1.33–1.21 (m, 22H), 0.88 (t, *J* = 7.0 Hz, 3H).

### 2.3. Synthesis of PCDA–Gabazine (**1**)

**Scheme 2:**
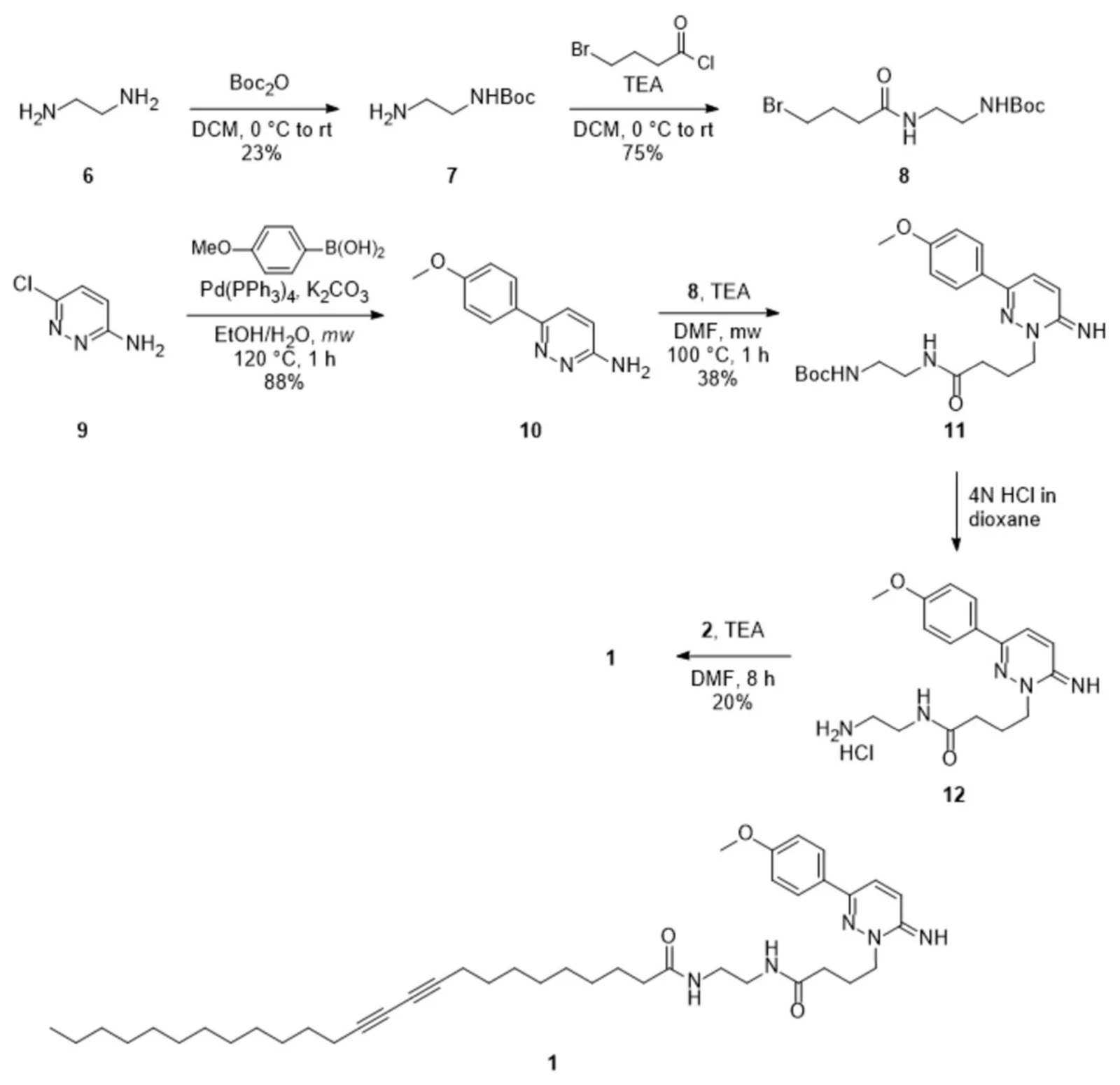
Synthesis of the PCDA–gabazine conjugate (**1**).

The gabazine fragment was prepared by adapting a reported microwave-assisted protocol [15] and used in this work only as a synthetic intermediate toward the PCDA–gabazine conjugate **1**, not as an isolated reagent. PCDA–gabazine (**1**) was assembled by coupling the activated PCDA–NHS intermediate **2** with a deprotected gabazine–ethylenediamine fragment **12** (Scheme 2). Briefly, ethylenediamine was mono-Boc-protected to give **7**, which was acylated with 4-bromobutanoyl chloride in the presence of triethylamine to afford **8**. In parallel, 3-amino-6-chloropyridazine (**9**) and 4-methoxyphenylboronic acid underwent a Pd-catalyzed Suzuki coupling under microwave irradiation to give 6-(4-methoxyphenyl)pyridazin-3-amine (**10**). Selective *N*(2)-alkylation of **10** with **8** under microwave irradiation furnished **11**, which was treated with 4 N HCl in dioxane to afford the amine hydrochloride salt **12**. Final amide coupling of **12** with PCDA–NHS (**2**) in the presence of triethylamine delivered the target conjugate **1**.

#### 2.3.1. *tert*-Butyl(2-aminoethyl)carbamate (**7**)

Di-*tert*-butyl dicarbonate (1.00 g, 4.7 mmol) was added to a solution of ethylenediamine (**6**) (2.00 g, 32.9 mmol) in DCM (20 mL) at 0 °C. The mixture was stirred for 24 h at room temperature and then filtered to remove a white precipitate. The filtrate was washed with water, dried over anhydrous MgSO_4_, filtered, and concentrated in vacuo to afford **7** as a colorless oil (1.00 g, 23% based on Boc_2_O). ^1^H NMR (600 MHz, CDCl_3_) δ 4.85 (s, 1H), 3.18 (q, *J* = 5.9 Hz, 2H), 2.80 (t, *J* = 5.9 Hz, 2H), 1.45 (s, 9H).

#### 2.3.2. *tert*-Butyl(2-(4-bromobutanamido)ethyl)carbamate (**8**)

4-Bromobutanoyl chloride (400 mg, 3.12 mmol) was added dropwise, followed by triethylamine (0.45 mL, 3.2 mmol), to a solution of **7** (345 mg, 3.12 mmol) in DCM (10 mL) at 0 °C. After stirring for 1 h, the reaction was quenched with water. The organic layer was separated, washed with brine, dried over anhydrous MgSO_4_, filtered, and concentrated in vacuo. The residue was purified by silica gel chromatography (DCM/MeOH, 25:1) to give **8** as a white solid (500 mg, 75%). ^1^H NMR (600 MHz, CDCl_3_) δ 6.40 (s, 1H), 5.01 (t, *J* = 6.1 Hz, 1H), 3.59 (t, *J* = 6.3 Hz, 2H), 3.35 (q, *J* = 5.6 Hz, 2H), 3.26 (q, *J* = 5.9 Hz, 2H), 2.35 (t, *J* = 7.2 Hz, 2H), 2.10 (p, *J* = 6.7 Hz, 2H), 1.43 (s, 9H).

#### 2.3.3. 6-(4-Methoxyphenyl)pyridazin-3-amine (**10**)

3-Amino-6-chloropyridazine (**9**) (300 mg, 2.3 mmol), K_2_CO_3_ (480 mg, 3.4 mmol), 4-methoxyphenylboronic acid (419 mg, 2.76 mmol), Pd(PPh_3_)_4_ (132 mg, 5 mol %), and EtOH/H_2_O (4:1, 5 mL) were combined in a sealed microwave vial. The mixture was degassed for 5 min, sealed, and irradiated at 120 °C for 1 h. The reaction mixture was filtered through Celite, the filtrate was concentrated, and the residue was purified by silica gel chromatography (DCM/MeOH, 20:1) to afford **10** as a white solid (410 mg, 88%). ^1^H NMR (600 MHz, DMSO-*d*_6_) δ 7.90–7.87 (m, 2H), 7.73 (d, *J* = 9.2 Hz, 1H), 7.02–6.99 (m, 2H), 6.81 (d, *J* = 9.3 Hz, 1H), 6.35 (s, 2H), 3.79 (s, 3H).

#### 2.3.4. *tert*-Butyl(2-(4-(6-imino-3-(4-methoxyphenyl)pyridazin-1(6H)-yl)butanamido)ethyl)carbamate (**11**)

Compound **10** (260 mg, 1.3 mmol), **8** (400 mg, 1.3 mmol), and DMF (2 mL) were combined in a sealed microwave vial and irradiated at 100 °C for 1 h. After cooling to room temperature, the solvent was evaporated. The residue was purified by silica gel chromatography (DCM/MeOH, 15:1) to give **11** as a white solid (210 mg, 38%). ^1^H NMR (600 MHz, DMSO-*d*_6_) δ 8.32 (d, *J* = 9.6 Hz, 1H), 7.96 (t, *J* = 5.7 Hz, 1H), 7.93–7.90 (m, 2H), 7.57 (d, *J* = 9.5 Hz, 1H), 7.12–7.07 (m, 2H), 6.76 (t, *J* = 5.8 Hz, 1H), 4.28 (t, *J* = 7.2 Hz, 2H), 3.82 (s, 3H), 3.00 (q, *J* = 6.3 Hz, 2H), 2.92 (q, *J* = 6.4 Hz, 2H), 2.24 (t, *J* = 7.1 Hz, 2H), 2.04 (p, *J* = 7.1 Hz, 2H), 1.34 (s, 9H).

#### 2.3.5. N-(2-Aminoethyl)-4-(6-imino-3-(4-methoxyphenyl)pyridazin-1(6H)-yl)butanamide hydrochloride (**12**)

Compound **11** (200 mg, 0.46 mmol) was dissolved in 4 N HCl in dioxane (3 mL) and stirred at room temperature for 15 min. The solvent was removed in vacuo to give the crude amine hydrochloride **12**, which was used in the next step without further purification. ^1^H NMR (600 MHz, DMSO-*d*_6_) δ 9.49 (s, 1H), 9.04 (s, 1H), 8.41–8.36 (m, 1H), 8.29–8.21 (m, 2H), 8.01–7.91 (m, 4H), 7.71 (dd, *J* = 9.6, 2.5 Hz, 1H), 7.11 (dq, *J* = 9.7, 2.3 Hz, 2H), 4.34 (td, *J* = 7.2, 2.5 Hz, 2H), 3.83 (d, *J* = 2.1 Hz, 3H), 3.26–3.20 (m, 2H), 2.80 (ddt, *J* = 11.2, 7.6, 4.1 Hz, 2H), 2.31 (td, *J* = 7.3, 2.5 Hz, 2H), 2.10–2.03 (m, 2H).

#### 2.3.6. PCDA–Gabazine (**1**)

PCDA–NHS (**2**) (152 mg, 0.32 mmol) was added to a solution of **12** (130 mg, 0.32 mmol) and triethylamine (0.07 mL, 0.48 mmol) in DMF (1.5 mL). The mixture was stirred overnight at room temperature. The solvent was removed in vacuo, and the residue was purified by silica gel chromatography (DCM/MeOH, 15:1) to give **1** as a white solid (45 mg, 20%). ^1^H NMR (600 MHz, CDCl_3_) δ 9.99 (s, 1H), 8.34 (dd, *J* = 9.5, 2.8 Hz, 1H), 8.09 (s, 1H), 7.91 (dd, *J* = 9.5, 2.7 Hz, 1H), 7.83–7.78 (m, 2H), 7.55 (s, 1H), 7.05–7.00 (m, 2H), 4.45 (t, *J* = 7.1 Hz, 2H), 3.89 (d, *J* = 3.0 Hz, 3H), 3.41 (s, 4H), 2.55 (d, *J* = 7.1 Hz, 2H), 2.30–2.26 (m, 2H), 2.22 (qd, *J* = 7.2, 2.3 Hz, 5H), 1.62 (s, 6H), 1.49 (tdd, *J* = 15.1, 8.1, 5.7 Hz, 5H), 1.39–1.20 (m, 24H), 0.88 (td, *J* = 7.0, 2.7 Hz, 3H).

### 2.4. Preparation of the GHB Detection Mat

The sensor mat was fabricated by multi-nozzle electrospinning, adapting a published protocol [16]. A matrix solution was prepared by dissolving cellulose and poly(vinylidene fluoride-*co*-hexafluoropropylene) (PVDF–HFP) in DMF at a cellulose: PVDF–HFP = 8:2 (*w*/*w*) ratio. To this matrix solution, PCDA and PCDA–EDA (**5**) were added at a PCDA: PCDA–EDA = 4:1 (*w*/*w*) ratio to give the final electrospinning dope. The dope was loaded into a 15 mL syringe fitted with a 22-gauge metal needle and processed on a multi-nozzle electrospinning apparatus (ESR200RD, NanoNC Co., Seoul, Korea) for 30 min at a flow rate of 12 mL h^−1^, an applied voltage of 25 kV, and a tip-to-collector distance of approximately 18.5 cm.

The nanofibers were collected on a rotating drum collector wrapped with aluminum foil to form a paper-like mat. In this manuscript, the term “paper-based sensor” refers to the cellulose-containing electrospun nanofiber mat used as the supporting substrate, rather than to conventional cellulose paper. The mat was dried at room temperature in the dark for at least 6 h, then cut into ~1 cm circular disks. Each disk was irradiated with a 254-nm UV lamp for 30 s to photopolymerize PCDA, generating the characteristic blue color of polydiacetylene (PDA). Successful photopolymerization was consistently achieved within the 10–60 s irradiation range, and 30 s was selected as the optimal condition throughout this study. The fabricated sensor was intended for single-use applications, as the blue-to-red color transition was irreversible under the experimental conditions used in this study.

### 2.5. GHB Detection Test

GHB test solutions were freshly prepared in distilled water at concentrations of 0.5, 1, 3, and 5% (*w*/*v*), where % (*w*/*v*) is defined as the mass of GHB in grams per 100 mL of the final solution. These concentrations correspond to 5, 10, 30, and 50 mg mL^−1^, respectively. Distilled water alone served as the 0% control. A 10 µL aliquot of each solution was spotted onto the center of a polymerized sensor disk and allowed to react for 30 s at room temperature. For the substituent-screening experiment, sensor disks containing PCDA–amide (**3**), PCDA–hydrazide (**4**), or PCDA–EDA (**5**) were separately exposed to 10 µL of 1% (*w*/*v*) GHB under identical experimental conditions. R-channel values were recorded at the standardized 30-s time point under the measurement conditions described in Section 2.6 and used to calculate ΔR.

### 2.6. Imaging Conditions

Sensor disks were analyzed using a Samsung Galaxy S23 smartphone (Samsung Electronics Co., Ltd., Suwon, Republic of Korea) equipped with a 50 MP, 1/1.56-inch CMOS rear camera (f/1.8, dual-pixel phase-detection autofocus, and optical image stabilization). Measurements were performed at a fixed working distance under uniform illumination from a 5500 K LED light source, with the sensor disks placed on a uniform white background and the smartphone mounted on a tripod. Under these conditions, the color-reproduction uncertainty inherent to the camera was estimated to be 5–10 RGB units (8-bit channels, 0–255), representing the instrumental error margin. The smartphone camera was positioned over the reaction zone before sample application, and the red-channel (R) value displayed in real time by the Color Meter mobile application (version 2.6.0; Contechity AB, Gothenburg, Sweden) was recorded at the standardized 30-s time point. The R-channel response to GHB was used as a semi-quantitative descriptor of the chromatic transition. Because the present work characterizes the device as a visual screening tool rather than a quantitative analytical assay, detection-limit metrics (LOD, LOQ) and reproducibility statistics across large replicate sets were not formally derived; their establishment is identified as a priority for the next stage of development (Section 3.5).

## 3. Results and Discussion

### 3.1. Synthesis of PCDA Derivatives and the PCDA–Gabazine Conjugate

To enable a controlled comparison of linker substituents, we adopted a modular synthetic strategy in which the activated PCDA–NHS ester (**2**) served as a common late-stage intermediate, diverging into the three derivatives **3**–**5**. EDC/NHS-mediated activation of PCDA afforded **2** in 78% yield. From **2**, two parallel amide-bond formations gave the targets directly. Treatment with hydrazine hydrate in DCM at room temperature furnished PCDA–hydrazide (**4**) in 51% yield. The corresponding ethylenediamine adduct (**5**, PCDA–EDA) was obtained in 67% yield by adding **2** dropwise to a large excess (~10 equiv) of ethylenediamine; this excess was essential to suppress bis-acylation of EDA and to drive selective mono-functionalization, leaving one free primary amine for evaluation in the subsequent substituent-screening experiment. PCDA–amide (**3**) was prepared from PCDA via the acyl chloride (oxalyl chloride/cat. DMF) followed by aqueous ammonia quench, giving **3** in 46% yield over two steps. All three derivatives **3**–**5** were characterized by ^1^H NMR.

The PCDA–gabazine conjugate (**1**) was assembled by amide coupling between PCDA–NHS (**2**) and the deprotected gabazine–ethylenediamine fragment (**12**), prepared in five steps from commercial starting materials (Scheme 2). Two design choices in this sequence merit comment. First, mono-Boc protection of ethylenediamine (**6** → **7**) was achieved by using Boc_2_O at a deliberately substoichiometric ratio (0.14 equiv relative to EDA); although this lowered the isolated yield of **7** to 23%, it effectively suppressed the bis-Boc by-product and gave clean mono-protected EDA after a single filtration of the precipitated bis-Boc material, eliminating the need for chromatographic separation at this stage. Second, the pyridazine ring of **10** offers two competing alkylation sites: the exocyclic 3-amino group and the endocyclic *N*(2) nitrogen. Under microwave irradiation in DMF at 100 °C, the alkylation with **8** proceeded with *N*(2)-regioselectivity, as indicated by the diagnostic downfield resonance of the pyridazine H4 (δ 8.32, d, *J* = 9.6 Hz) and the persistence of the exocyclic NH_2_ signal (δ 7.96, t) in the ^1^H NMR spectrum of **11**, consistent with reported gabazine-type analogues [15].

Microwave heating was used in two key steps. The Pd-catalyzed Suzuki coupling between 3-amino-6-chloropyridazine (**9**) and 4-methoxyphenylboronic acid was completed in 1 h at 120 °C (88% yield for **10**), and the subsequent *N*(2)-alkylation was carried out in 1 h at 100 °C (38% yield for **11**); both transformations were substantially faster than conventional thermal protocols. After Boc removal with 4 N HCl in dioxane (giving **12**, used directly without purification), final amide coupling with PCDA–NHS (**2**) and triethylamine in DMF delivered the target conjugate **1** in 20% yield as a white solid. While the moderate yield of the final amide coupling reflects the limited solubility of the polar amine salt **12** in DMF, the overall route nevertheless provides reproducible access to **1** on a sub-millimole scale, securing a reliable supply of this benchmark compound for the comparative studies below.

### 3.2. Substituent Screen Identifies EDA as the Optimal Linker

With the three derivatives **3**–**5** in hand, we benchmarked each against 1% *w*/*v* aqueous GHB and recorded the colorimetric response (ΔR) at 30 s. Representative single-measurement responses are summarized in Table 1.

The amide derivative **3**, which lacks any pendant nucleophilic nitrogen, produced only a marginal response (ΔR = 12), consistent with the absence of a recognition handle. Introducing a hydrazide nitrogen (**4**) increased the response approximately three-fold (ΔR = 34), and replacing it with the ethylenediamine bridge (**5**) gave a further roughly two-fold increase (ΔR = 53). At the standardized 30 s time point, PCDA–EDA exhibited the strongest response among the three derivatives. The clear monotonic ordering—3 ≪ 4 < 5—and the rapid visual response of 5 identify PCDA–EDA as the optimal substituent and motivate its use in all subsequent device-level work. This trend is consistent with general observations that primary aliphatic amines outperform less nucleophilic nitrogen functionalities in PDA chromism [13].

### 3.3. Visual Screening Performance of the PCDA–EDA Sensor Mat

**Figure 2:**
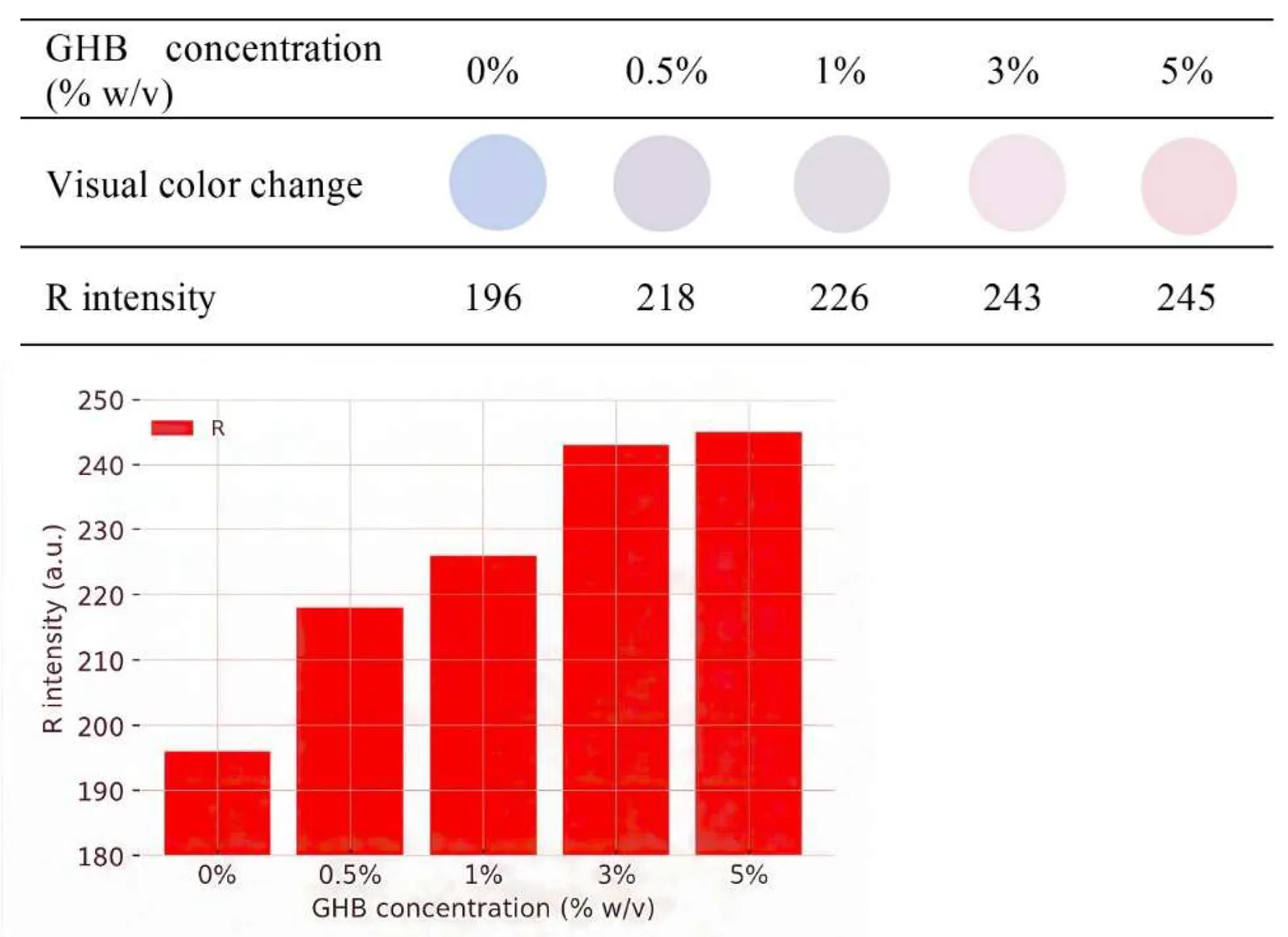
Concentration-dependent R-channel intensity of the PCDA–EDA sensor recorded 30 s after exposure to GHB (single representative measurement at 0, 0.5, 1, 3, and 5% (*w*/*v*)).

Building on the mechanistic findings, we characterized the visual response of the PCDA–EDA paper sensor against aqueous GHB across the 0–5% (*w*/*v*) range. All R-channel intensities presented in Figure 2 were recorded at the standardized 30 s time point. The R-channel intensity (single representative measurement at each concentration) increased monotonically with GHB concentration, from 196 (0%, blank) to 218 (0.5%), 226 (1%), 243 (3%), and 245 (5%) (Figure 2). The response was clearly distinguishable from the blank at 0.5% *w*/*v* (≈5 mg mL^−1^ ≈ 40 mM)—which corresponds to the typical clandestine dose of 1–2 g GHB in a 200 mL beverage (≈0.5–1% *w*/*v*) [1]—placing the operational range of the device within the forensically relevant window.

Table 1 and Figure 2 were generated from independent experimental series conducted for different purposes. Table 1 presents a side-by-side comparison of the three PCDA derivatives, whereas Figure 2 presents the concentration-dependent response of PCDA–EDA. In both experiments, the R-channel values were recorded at 30 s. The ΔR value in Table 1 was calculated using the matched blank from the same substituent screening experiment, whereas Figure 2 presents absolute R-channel intensities from a separate concentration series experiment. Therefore, the absolute intensity values in Figure 2 should not be used to recalculate the ΔR value reported in Table 1.

The response curve flattened above 3% *w*/*v*, with the increment from 3% to 5% (ΔR = 2) falling within the camera’s intrinsic color-reproduction uncertainty (5–10 RGB units). This response saturation is consistent with the progressive occupation of the accessible EDA recognition sites in the polymerized PDA backbone; once a substantial fraction of the binding pockets is engaged, further ligand addition cannot drive additional conformational rearrangement of the ene-yne system. The visually useful range of the sensor therefore spans ~0.5–3% *w*/*v* (~5–30 mg mL^−1^), with a monotonic chromatic progression suitable for semi-quantitative concentration estimation by eye or by smartphone-based image analysis.

For direct comparison, a PCDA–gabazine mat prepared using the same fabrication protocol was exposed to 0.5–5% (*w*/*v*) GHB. No visually detectable blue-to-red transition occurred within the standard 30 s response time. Therefore, this comparison was not subjected to quantitative ΔR analysis. Instead, photographs of the actual test sheets taken approximately 24 h after GHB exposure are provided in Figure S2 as a qualitative comparison. Even after this prolonged exposure, the mats remained predominantly blue without a visually appreciable color transition.

### 3.4. Material and Process Optimization

Beyond the choice of recognition chemistry, the matrix and fabrication route govern reproducibility and throughput. The conventional protocol—single-nozzle electrospinning of a PVDF–HFP/poly(ethylene oxide) (7:3) matrix loaded with PCDA:gabazine (4:1) in NMP/acetone—required ~120 min per sheet, gave variable fiber diameters, and was therefore poorly suited for scale-up [17]. We reformulated the dope to a cellulose: PVDF–HFP (8:2, *w*/*w*) matrix dissolved in DMF, with a PCDA:PCDA–EDA (4:1, *w*/*w*) sensing pair, and processed it on a multi-nozzle apparatus (Table 2).

Three changes were responsible for the improvement. (i) Replacing PEO with cellulose increased the matrix porosity and water-wettability, accelerating analyte transport to the embedded recognition layer. (ii) Switching the solvent system from NMP/acetone to DMF gave a single, slower-evaporating phase that stabilizes the electrospinning jet and narrows the fiber-diameter distribution; the disappearance of macroscopic beading observed under the conventional protocol is consistent with this observation [18,19]. (iii) Raising the applied voltage from 20 to 25 kV brought the Taylor cone into a stable jetting regime at the higher mass throughput required by the multi-nozzle head, where four parallel nozzles deposit fibers on a common rotating drum [20,21]. Together, these changes reduced fabrication time approximately four-fold, from ~120 min per sheet to ~30 min per sheet, while improving fiber uniformity. The other process parameters—tip-to-collector distance (~18.5 cm), drum collection, and 254-nm UV polymerization (10–60 s)—were retained from the conventional protocol.

### 3.5. Practical Considerations and Limitations

Several limitations of this study should be acknowledged before forensic deployment. First, because the present work characterizes the device as a visual screening tool, quantitative analytical figures of merit (formal LOD, LOQ, calibration linearity statistics, and replicate reproducibility) were not derived in this study. Establishing these metrics—with full replicate sets at each concentration and a properly characterized blank distribution—is the most important next step toward routine analytical use. Second, although the sensor response was evaluated using ten GHB-spiked beverage matrices, with ten independent measurements per matrix (total *n* = 100; Figure S1), the evaluation was limited to a single GHB concentration under controlled conditions. Third, imaging was performed under controlled illumination using a single smartphone camera; field variability in lighting, white balance, temperature, and humidity may shift the absolute R-channel intensities, and a mobile-application-based per-image white-point calibration is the natural mitigation [21]. Fourth, the chromatic transition is intrinsically a one-way blue-to-red event, and the device is therefore single-use. This is acceptable for the intended forensic screening scenario but should be communicated clearly on any deployed kit.

These limitations notwithstanding, the combination of the mechanistically targeted PCDA–EDA chemistry, the cellulose/PVDF–HFP/DMF matrix, and multi-nozzle fabrication delivers a fast, low-cost, instrument-free visual readout and a fabrication route compatible with batch production—the principal practical requirements for an at-the-glass GHB screening device.

## 4. Conclusions

In this work, we reassigned the molecular origin of GHB recognition in polydiacetylene (PDA) colorimetric sensors. To our knowledge, this is the first study to provide a direct experimental comparison showing that the ethylenediamine (EDA) linker plays a more important role in GHB recognition than the gabazine pharmacophore in PCDA-conjugate sensors. A side-by-side screen of three PCDA derivatives ranked the colorimetric response within 30 s as PCDA–EDA (ΔR = 53) > PCDA–hydrazide (ΔR = 34) ≫ PCDA–amide (ΔR = 12), whereas a PCDA–gabazine mat prepared by the same fabrication protocol showed only a subtle, barely discernible color shift after several hours and remained predominantly blue even after approximately 24 h, without a visually appreciable blue-to-red transition. The substituent screen, together with the markedly slower and weaker response of PCDA–gabazine, supports the conclusion that the EDA linker plays a more important role in GHB recognition than the gabazine pharmacophore. These experimental results suggest that the accessible free primary amine of PCDA–EDA contributes to its faster and stronger response under the tested conditions.

However, the precise molecular interaction responsible for this difference was not determined in the present study and warrants further investigation.

Building on this mechanistic finding, we developed a paper-based sensor using a modified matrix formulation and a four-nozzle electrospinning process. Under the current cellulose/PVDF–HFP/DMF formulation, stable and continuous electrospinning was maintained, and the fabrication time was reduced from approximately 120 to 30 min per sheet, corresponding to a 75% reduction in processing time. The resulting mats produced a clearly distinguishable, dose-dependent visual blue-to-red transition across the forensically relevant range of 0.5–3% *w*/*v* GHB (~5–30 mg mL^−1^) within 30 s, suitable as a rapid on-site visual screening device. In parallel, an improved synthetic route to the PCDA–gabazine conjugate (**1**) was established to secure a reliable supply of this benchmark compound for future comparative work.

Beyond the immediate device, the broader implication of this study is methodological: in PDA-based supramolecular sensors, the linker architecture is at least as decisive as the named pharmacophore in setting both selectivity and chromatic response [4]. We therefore recommend that future PDA-conjugate designs treat the linker not as a passive tether but as a co-equal design parameter. Formal analytical validation (LOD, LOQ, calibration statistics, replicate reproducibility), selectivity against common beverage interferents (ethanol, sugars, organic acids, caffeine), and expanded beverage-matrix validation across multiple GHB concentrations and under variable lighting remain important next steps toward field deployment.

## Figures and Tables

**Table 1:** Influence of the linker substituent on GHB detection performance (1% *w*/*v* aqueous GHB, 30 s; single representative measurement).

Sensor	Reaction Time	ΔR	Visual Response
PCDA–EDA (**5**)	30 s	53	Strong (blue → red)
PCDA–hydrazide (**4**)	30 s	34	Moderate (blue → purple)
PCDA–amide (**3**)	30 s	12	Negligible

**Table 2:** Comparison of the conventional single-nozzle and the optimized multi-nozzle electrospinning protocols.

Parameter	Conventional	Optimized (This Work)
Polymer matrix	PVDF–HFP:PEO = 7:3	Cellulose:PVDF–HFP = 8:2
Sensing pair	PCDA:gabazine = 4:1	PCDA:PCDA–EDA = 4:1
Solvent	NMP/acetone	DMF
Nozzle configuration	Single nozzle	Multi-nozzle (4 parallel)
Applied voltage	20 kV	25 kV
Spinning distance	~18.5 cm	~18.5 cm
UV polymerization	254 nm, 10–60 s	254 nm, 10–60 s
Fabrication time	~120 min/sheet	~30 min/sheet (4× faster)
Electrospinnability	Solidification-limited	Stable and continuous

## Data Availability

The data supporting the findings of this study are available within the article. Raw NMR spectra are available from the corresponding author upon reasonable request.

## Supplementary Materials

The following supporting information can be downloaded at https://www.mdpi.com/article/10.3390/bios16080440/s1.

## Abbreviations

ΔR	change in red-channel intensity
^1^H NMR	proton nuclear magnetic resonance
DCM	dichloromethane
DMF	*N*,*N*-dimethylformamide
EDA	ethylenediamine
EDC	1-ethyl-3-(3-dimethylaminopropyl)carbodiimide hydrochloride
GHB	γ-hydroxybutyric acid
NHS	*N*-hydroxysuccinimide
NMP	N-Methyl-2-pyrrolidone
PCDA	10,12-pentacosadiynoic acid (monomer)
PDA	polydiacetylene (UV-polymerized PCDA)
PVDF–HFP	poly(vinylidene fluoride-*co*-hexafluoropropylene)
TEA	triethylamine
