# Supplementary material for: PCDA–EDA Colorimetric Nanofiber Sensor for Rapid Visual GHB Screening: Linker Reassignment and Scalable Fabrication
Source: Biosensors (Basel). 2026 Aug 14;16(8):440. doi: 10.3390/bios16080440 (PMC13511193; doi:10.3390/bios16080440)
Supplement: Supplementary file 1 [file biosensors-16-00440-s001.zip › biosensors-4379539-supplementary.pdf]

Supporting Information

PCDA–EDA Colorimetric Nanofiber Sensor for Rapid Visual GHB Screening: Linker Reassignment and Scalable Fabrication

Seunghye Yang, Jeongwook Lee, Om Darlami * and Dongyun Shin *

College of Pharmacy, Gachon University, 191 Hambakmoe-ro, Yeonsu-gu, Incheon 21936, Republic of Korea; ljw7764@chemone.kr (J.L.)

* Correspondence: 21592103@gachon.ac.kr (O.D.); dyshin@gachon.ac.kr (D.S.)

Contents

1. ¹H-NMR of synthesized compounds
2. Evaluation of the PCDA sensor response in different beverage matrices
3. Evaluation of the PCDA–gabazine sensor response to GHB

1. ¹H-NMR of synthesized compounds

¹H NMR of compound **2** ($CDCl_3$)

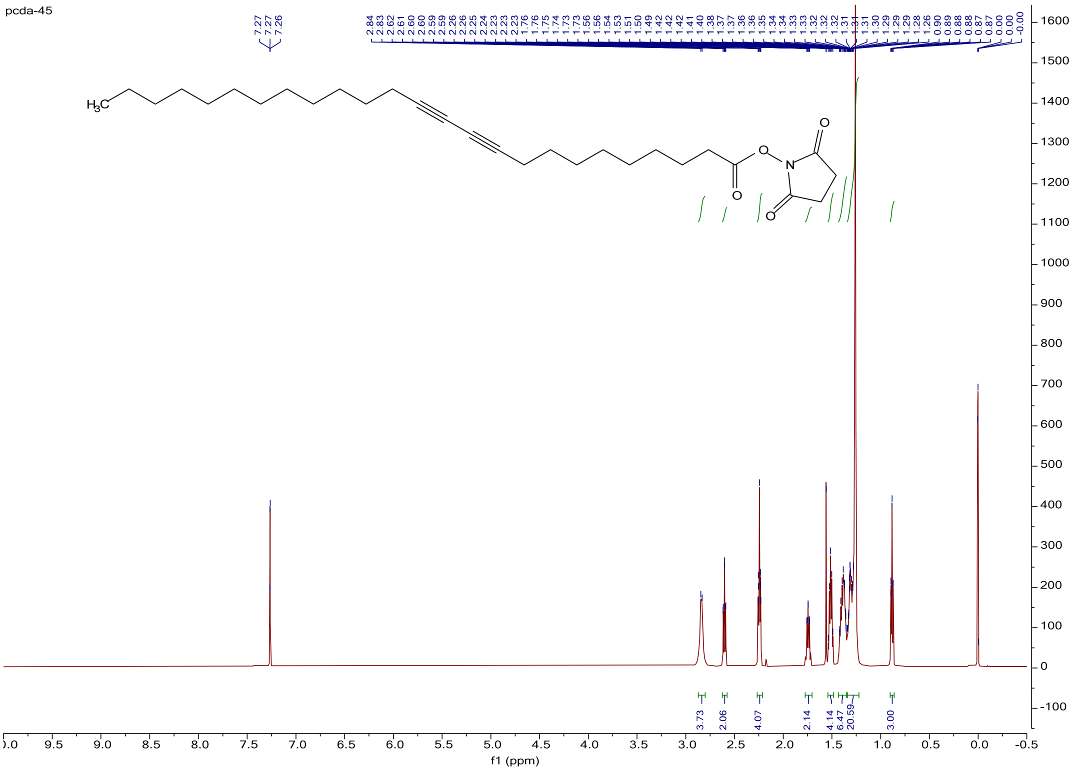

¹H NMR of compound **3** ($CDCl_3$)

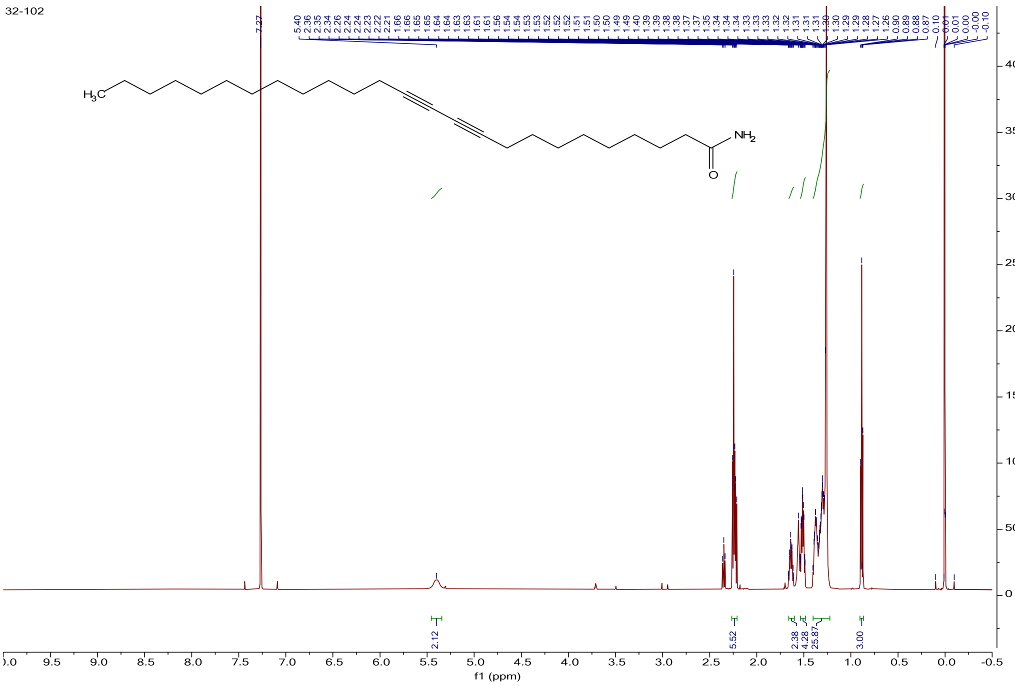

^{1}H NMR of compound **4** ($CDCl_3$)

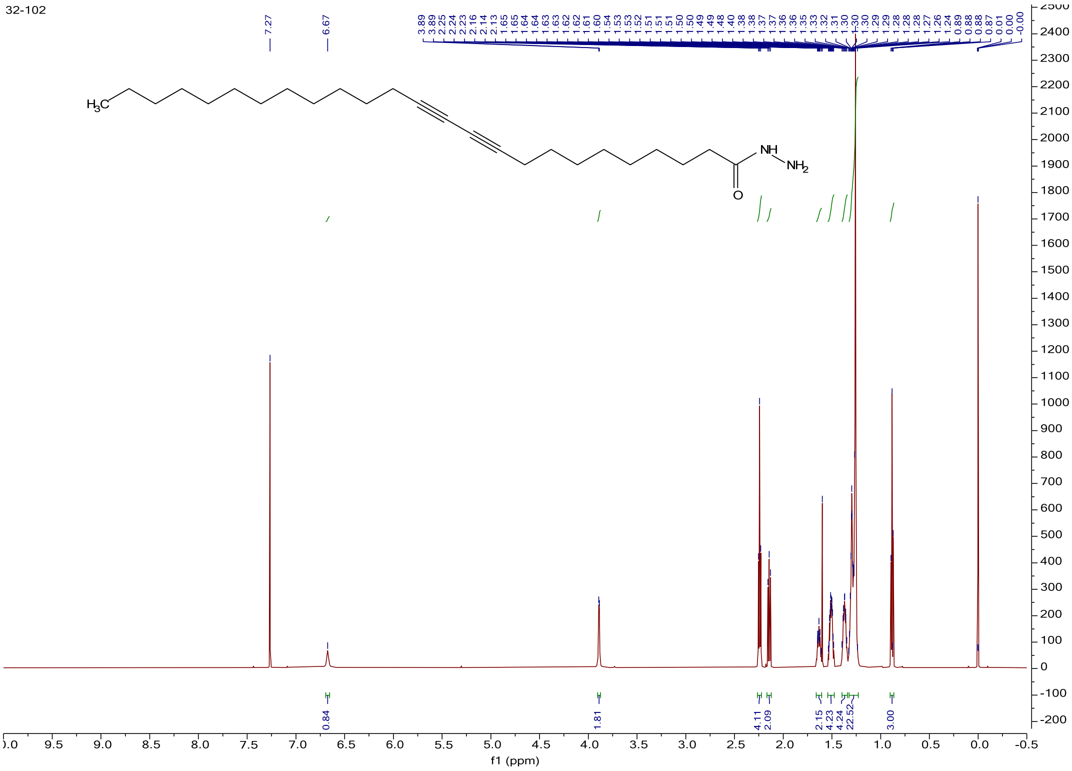

^{1}H NMR of compound **5** ($CDCl_3$)

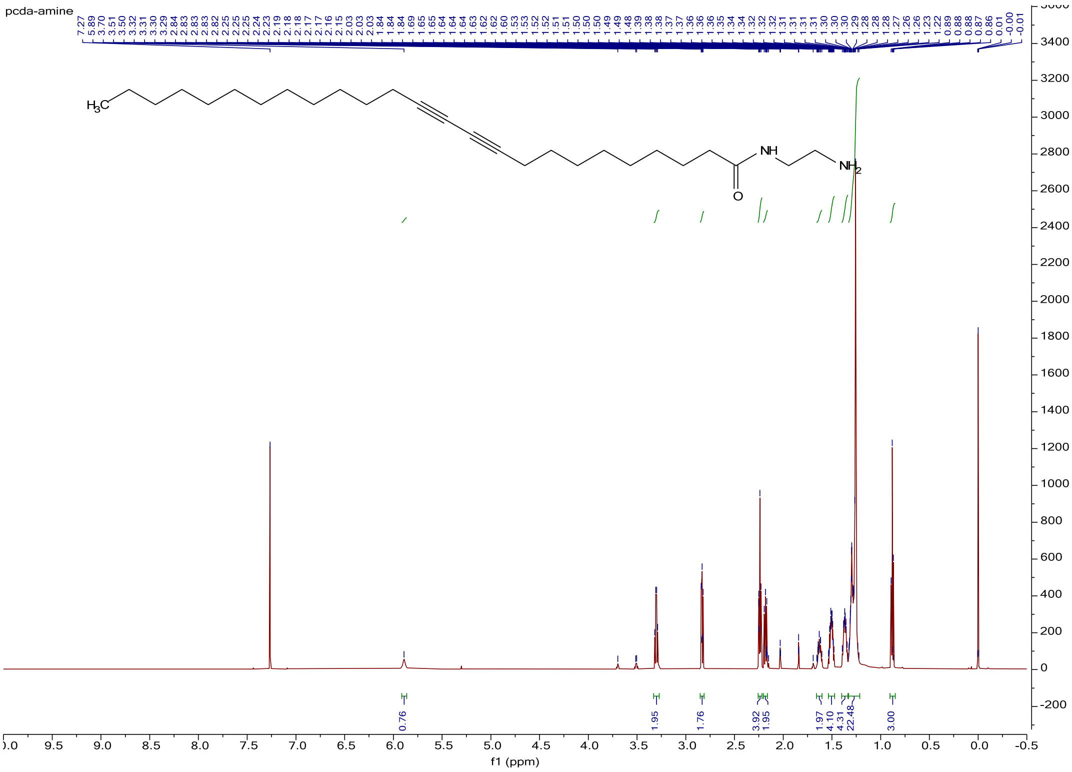

^{1}H NMR of compound **7** ($CDCl_3$)

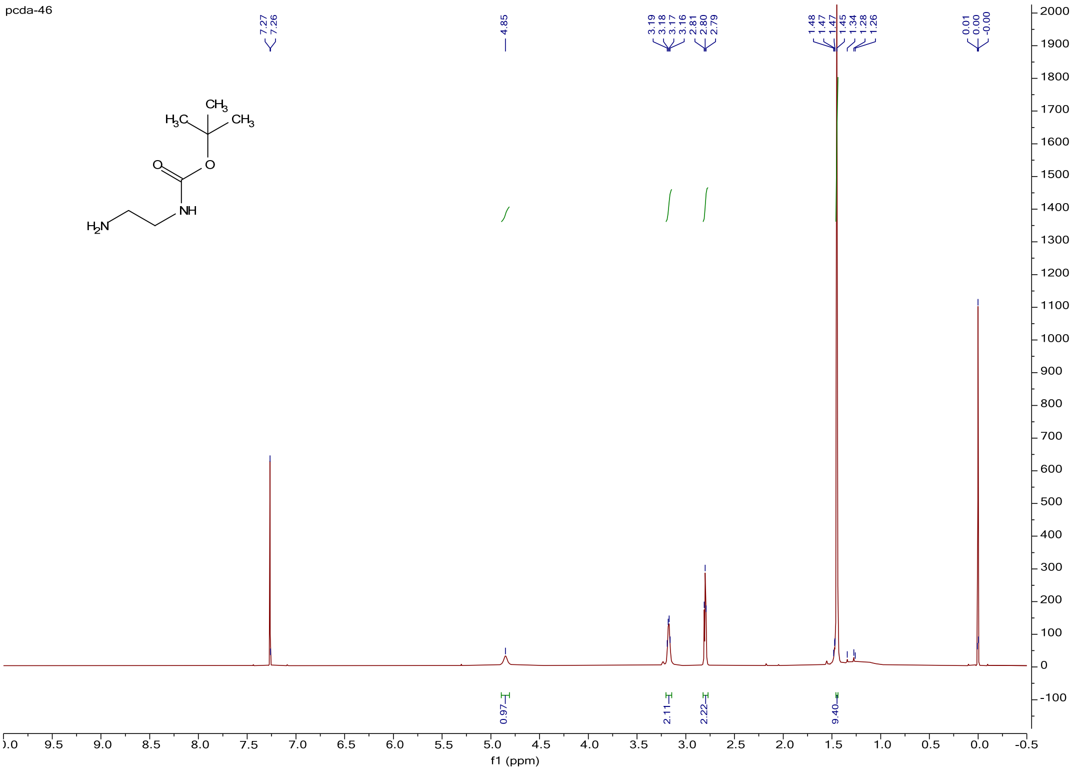

^{1}H NMR of compound **8** ($CDCl_3$)

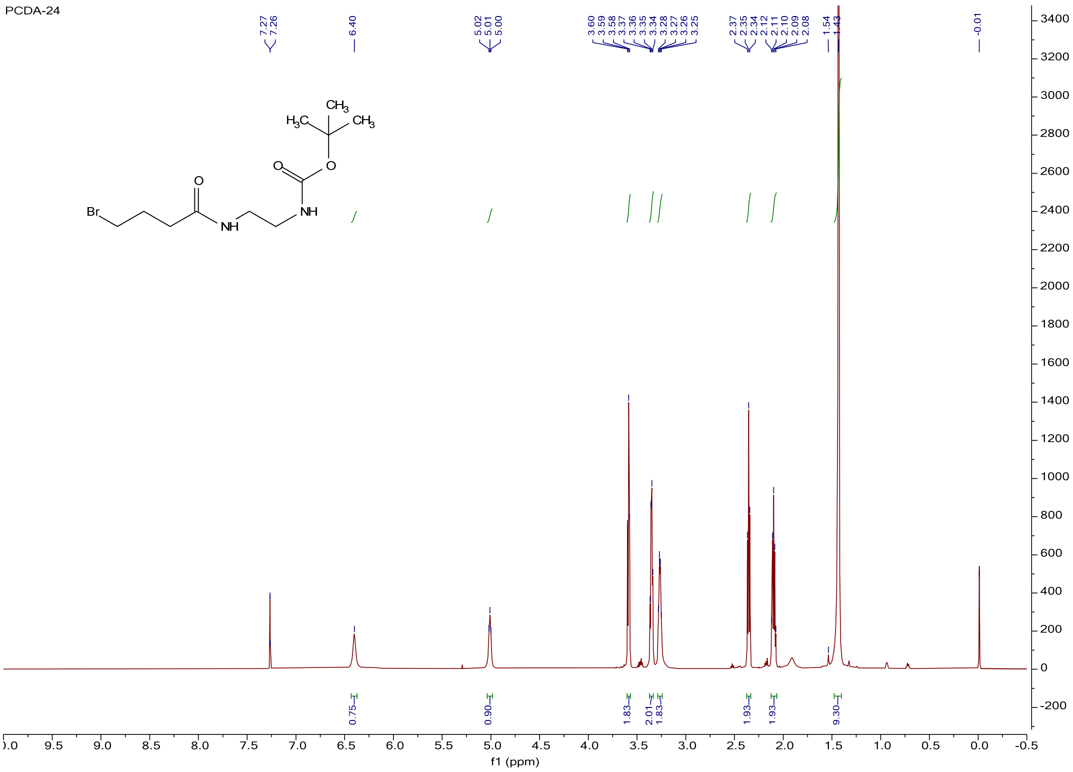

^{1}H NMR of compound **10** (DMSO-d_6)

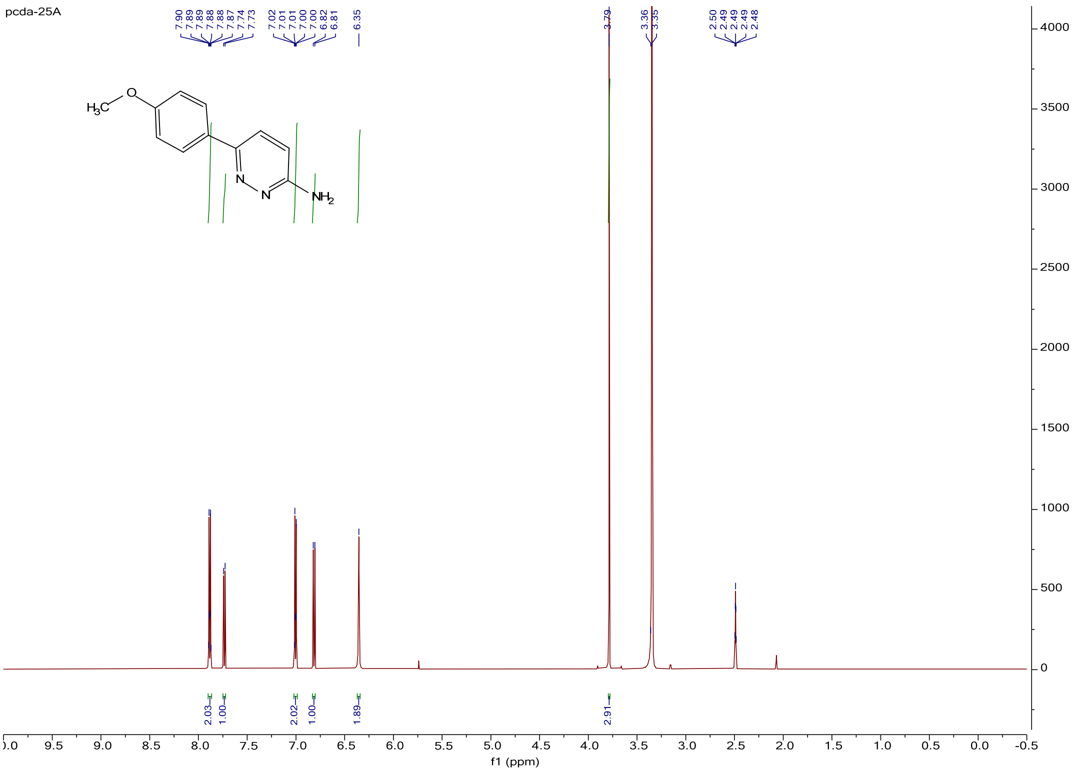

^{1}H NMR of compound **11** (DMSO-d_6)

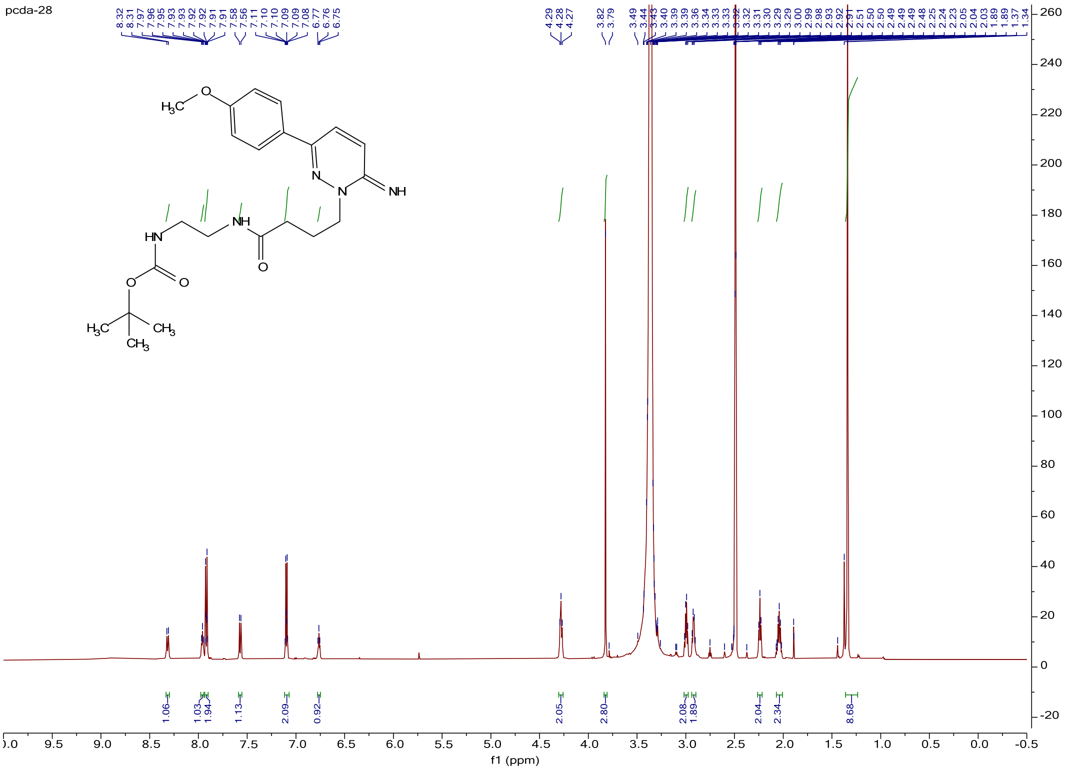

^{1}H NMR of compound **12** (DMSO-d_6)

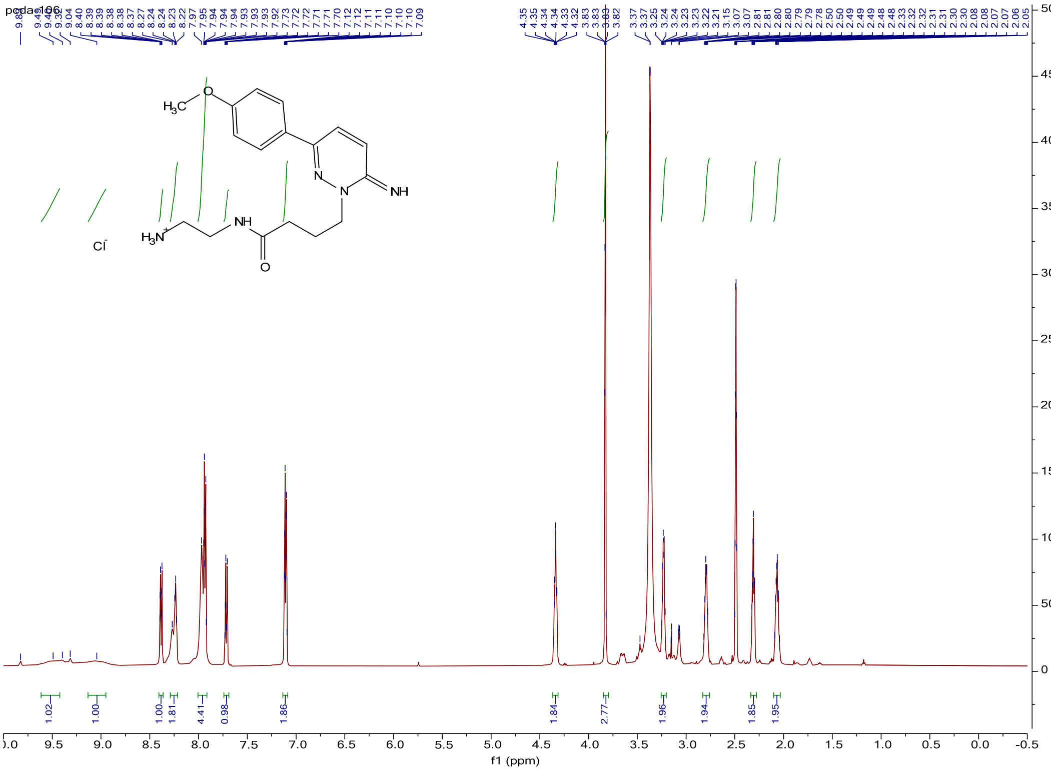

^{1}H NMR of compound **1** ($CDCl_3$)

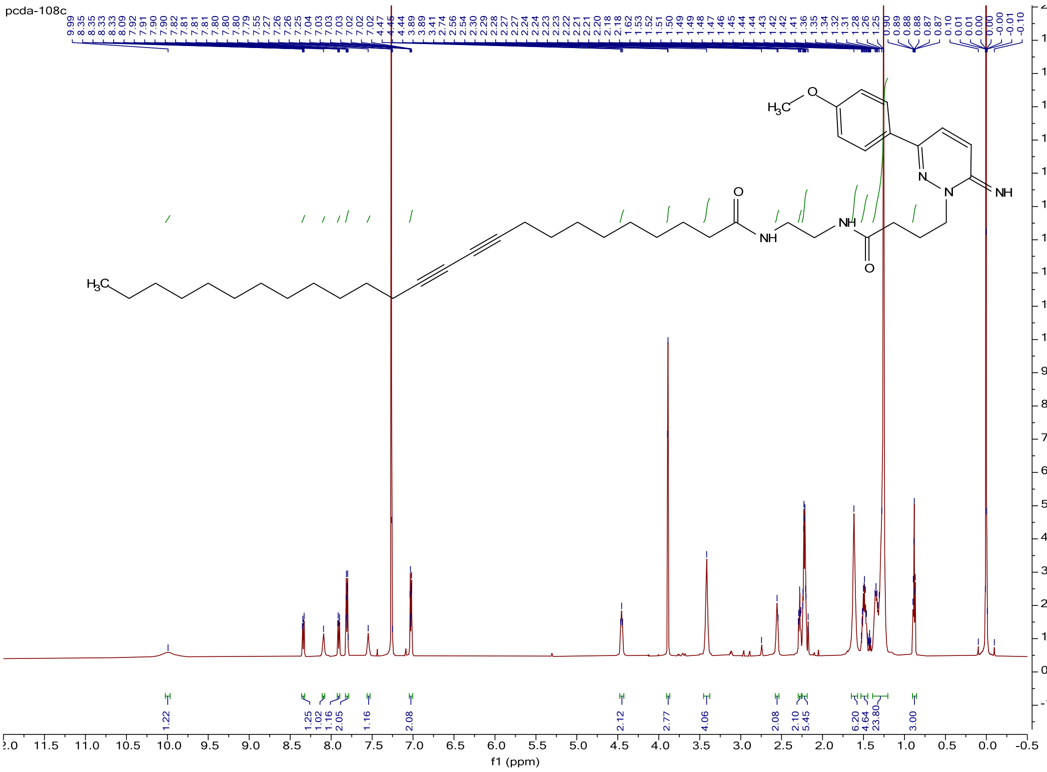

2. Evaluation of the PCDA sensor response in different beverage matrices

To evaluate the effect of beverage matrices on the colorimetric response of the PCDA sensor, GHB (sodium salt) was dissolved separately in ten different beverages (water, soju, beer, vodka, whiskey, coffee, milk, soft drink, sports drink, and fruit juice) at a final concentration of **0.5% (w/v)**. Twenty microliters of each sample was applied to the PCDA sensor, and the color change was recorded after the reaction. Ten independent measurements were performed for each beverage matrix (n = 10). Representative colors were extracted from the sensor images, and the average color for each beverage matrix was calculated to generate the color palette shown in Figure S1.

**Beverage Type**	None	Water	Soju	Beer	Vodka	Whiskey	Coffee	Milk	Soft Drink	Sport Drink	Fruit Juice
**GHB 0.5%(w/v)**											

Figure S1. Average representative colors of the PCDA sensor after exposure to GHB (0.5% (w/v)) in different beverage matrices. Each beverage was tested in 10 independent replicates (10 beverages; total n = 100). The displayed colors represent the average color for each beverage.

※ GHB (sodium salt) used in this study was legally imported and handled under approval from the Korean Ministry of Food and Drug Safety (MFDS) for academic research.

3. Evaluation of the PCDA–gabazine sensor response to GHB

To evaluate the response of the PCDA–gabazine sensor to GHB, aqueous GHB (sodium salt) solutions at concentrations of 0.5, 1, 3, and 5% (w/v) were applied separately to the sensor. The sensor images were acquired approximately 24 h after sample application. Despite the prolonged observation period, the sensor remained predominantly blue, with no visually appreciable blue-to-red color transition across the tested concentration range.

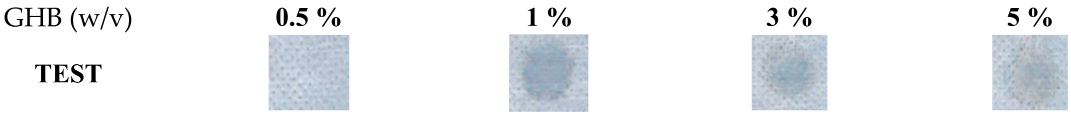

Figure S2. Representative visual responses of the PCDA–gabazine sensor following exposure to aqueous GHB solutions at concentrations of 0.5, 1, 3, and 5% (w/v). The images were acquired approximately 24 h after sample application. No visually appreciable blue-to-red color transition was observed across the tested concentration range, and the sensor remained predominantly blue.

※ GHB (sodium salt) used in this study was legally imported and handled under approval from the Korean Ministry of Food and Drug Safety (MFDS) for academic research.
